# Establishment and optimization of mRNA in situ hybridization system in turnip (*Brassica rapa* var*. rapa*)

**DOI:** 10.1186/s13007-019-0499-4

**Published:** 2019-10-16

**Authors:** Cheng Li, Simin Hu, Qidong Lei, Chongde Wang, Yunqiang Yang, Yongping Yang, Xudong Sun

**Affiliations:** 1 Key Laboratory for Plant Diversity and Biogeography of East Asia, Kunming Institute of Botany, Chinese Academy of Sciences, Kunming, 650201 China; 2 Plant Germplasm and Genomics Center, The Germplasm Bank of Wild Species, Kunming Institute of Botany, Chinese Academy of Sciences, Kunming, 650201 China; 3 University of Chinese Academy of Sciences, Beijing, China; 4 Faculty of Life Science and Technology, Kunming University of Science and Technology, Kunming, Yunnan China; 5 College of Plant Protection, Yunnan Agriculture University, Kunming, 650201 China

**Keywords:** mRNA in situ hybridization, *Brassica rapa* var*. rapa*, Gene expression, *BrrCLV3*, *BrrWUSa*

## Abstract

**Background:**

In situ hybridization (ISH) is a general molecular biological technique used to determine the spatiotemporal expression of genes in many species. In the past few years, numerous ISH protocols have been established in many species. Turnip (*Brassica rapa* var. *rapa*) is an important crop in the world, especially in the Plateau area of China, and is a traditional Tibetan medicine. However, ISH protocol in turnip has not been established.

**Results:**

We explored and established an optimal workflow for mRNA ISH system for turnip which has been evaluated using *BrrCLV3* and *BrrWUSa*. The optimal methods include: (1) fixation method, (2) protease K pretreatment time, (3) probe length and concentration, (4) washing temperature. We also provide advice on weakening background and improving the efficiency of RNA transcription in vitro. The expression of the *BrrCLV3* gene in turnip was detected by the optimized system, and the applicability of the system was confirmed by using *BrrWUSa*.

**Conclusions:**

In this study, we established and optimized the mRNA ISH system for turnip. We explored and found that (1) FAA fixative was the optimized fixation method, (2) 30 min was the optimized protease K pretreatment time, (3) 100 bp, 100 ng/ml probe had good hybridization signal, (4) the optimized washing temperature was 52 °C. It provides a powerful method to locate mRNA in the tissue, which can study the expression and function of turnip’s genes. As such, it has considerable advantages in terms of time and cost.

## Background

In situ hybridization (ISH) was established in 1969 by Gall and John et al. [1, 2]. ISH is a molecular biological technique for detecting the location of DNA in chromosomes or mRNA expression in tissues at the cellular level [3]. In situ hybridization has broad range of applications and has been used to (a) localize viral infection, (b) identify sites of gene expression, (c) analyze mRNA transcription and tissue distribution, and (d) map gene sequences in chromosomes [4]. mRNA ISH is an important tool for detecting mRNA expression sites and analyzing gene expression and regulation in tissues. Genes demonstrate spatiotemporal expression specificity during plant growth and development. Therefore, studying the mechanism of plant growth and development by detecting the expression patterns of genes in different genetic backgrounds, developmental stages, and environments is important. ISH is a general molecular biological technique used to determine the spatiotemporal expression of genes. Recent years, ISH protocol has been established and used in *Medicago truncatula* [5], *Zea mays* [6], *Kalanchoë daigremontiana* [7] and rust fungi in plant tissue sections [8] to research gene expression.

Turnip (*Brassica rapa* var. *rapa*) is a biennial plant belonging to Cruciferae. It’s an important economic crop in various regions of China. The tuber roots and leaves of turnip are edible and have high nutritional value because of their abundant proteins, fatty acids, flavonoids, amino acids, and other substances [9]. As a traditional Tibetan medicine, turnip seeds and tubers can be used as medicine because of their high medicinal value [10]. Moreover, many researchers have also confirmed turnip’s medicinal value. It can reduce the risk of liver fibrosis [11], inhibit the activity of human breast and colon cancer cells [12], increase the body’s ability to withstand hypoxia [13]. However, gene expression pattern is difficult to detect due to a lack of suitable transgenic method in turnip. Because of the complexity of the procedure and the variation between different plant tissues and probe sequences [14]. Therefore, it has great significance to established ISH protocol of turnip, in order to discover the gene expression patterns in turnip.

## Methods

### Experimental materials

*Brassica rapa* var. *rapa* KTRG-B-54 was obtained from Ninglang County, Lijiang City, Yunnan Province, China.

### Experimental methods

#### Cloning of *BrrCLV3* and *BrrWUSa* genes

Primers were designed based on the *CLV3* and *WUS* gene sequences of Chinese cabbage and used for cloning *BrrCLV*3 (GenBank accession number: MN481053) and *BrrWUSa* (GenBank accession number: MN481054) genes, respectively. The primer sequences are shown in Table 1.

**Table 1:** Primers for the amplification of full-length CDS of *BrrCLV3* and *BrrWUSa* gene

Gene	Forward primer	Reverse primer
*BrrCLV3*	5′ATGGATTCGAGGACTCTGGTGCT3′	5′TTAAGGGATATGAGAGTCGGTTCG3′
*BrrWUSa*	5′ATGGAGCCACCGCAACATCATCATC3′	5′ATCCTGTGTGACGCCGCGGCGTGGG3′

#### Designing of the probe

After sequencing *BrrCLV3* and *BrrWUSa*, we compared the genomic sequences of Chinese cabbage and designed probes (Table 2).

**Table 2:** Probe sequences of *BrrCLV3* and *BrrWUSa* genes in situ hybridization

Gene	Probe sequence
*BrrCLV3*	ATGGATTCGAGGACTCTGGTGCTACTGCTGCTCTTTTGCCTCATGTT CCTGCATGATGCTTCTGATATCACTCACGCCAATGCCAATGTTCATG CACTTCCCATTCGCAAGATGATGGTAATGAAGAAGGATAATGAATG GGGAGGAGCAAATGGAATTGAAGAAGAGAAGGAGAAGGTTTTCG GGTTAAATGAAGAACTAAGGACTGTCCCTTCAGGACCTGACCCTTT GCACCATCATGTGAACCCCCCAAGAAAGCCACGAACCCGACTCTCA TATCCCT
*BrrWUSa*	CGTACTGAAATAGGCTTAAATTCCATATTAATATAATTTCAATGTGTT ACATTATAAAAGAAAGGAGAAAAACACACTCGAATAAAGTTAAAC CTAGACGATAGTAATTACAATTTATTAAACTACGTACGTACGGAAGA TACTGCTATTACTGAATAATACGGGCCAACCATGCGAATATGCGATT GAAGCTAAAGAGCGTAGGAGTTCAGACGTAGCTCAAGAAAGTATT AATCCTGTGTGACGCCGGCGGCGTAGGAGTTCAGGCATAGCTTTAG AGAAGCACAAGGG

#### In vitro transcription of RNA probe

The probe sequences of *BrrCLV3* and *BrrWUSa* were cloned used the primers: *BrrCLV3*F and *BrrCLV3*R as well as *BrrWUSa*F and *BrrWUSa*R, respectively (Table 3). The plasmids were digested with *Nde*I or *Nco*I endonucleases of NEB (New England Biolabs, Beijing), respectively. We used two methods to transcribe the probes.

**Table 3:** Primers of in situ hybridization probe of *BrrCLV3* gene and *BrrWUSa* gene

Gene	Forward primer	Reverse primer
Probe of *BrrCLV3*	5′ATGGATTCGAGGACTCTGGTGCT3′	5′TTAAGGGATATGAGAGTCGGTTCG3′
Probe of *BrrWUSa*	5′CCCTTGTGCTTCTCTAAAGCTATGC3′	5′CGTACTGAAATAGGCTTAAATTCCA3′

1. Conventional method: After enzyme digestion, the ethanol product was recovered, transcribed as a template, and incubated at 37 °C for 2 h. The *Nde*I and *Nco*I products were transcribed using T7 RNA polymerase and SP6 RNA polymerase, respectively, to achieve sense and antisense probes.
2. Improved method: After enzyme digestion, the products were directly used as the template by adding the maximum amount of RNasin (Solarbo, Beijing, China) and RNA polymerase, and the incubation time increased to 5 h at 37 °C. The *Nde*I or *Nco*I products were transcribed using T7 RNA polymerase or SP6 RNA polymerase, respectively, to obtain sense and antisense probes.

#### Probe hydrolysis

The *BrrCLV3* and *BrrWUSa* probes were incubated at 60 °C for 59 min and 60 min to hydrolyze to the fragments approximately 100 bp long, respectively.

#### Detection of probe concentration

The probe concentration of digoxin (DIG)-labeled RNA was detected by dot blotting.

1. Added 1 µl labeled control RNA (100 ng/µl) to 99 µl diluent buffer (diethyl pyrocarbonate (DEPC) treated H_2_O:20× sodium citrate buffer (SSC):formaldehyde = 5:3:1) to obtain 1 ng/µl labeled control RNA.
2. Transferred 5 µl from this to 45 µl dilution buffer to obtain 100 pg/µl labeled control RNA.
3. Repeated step (2) to get the 10 pg/µl and 1 pg/µl labeled control RNA.
4. For the test RNA, added 1 µl RNA into the 9 µl dilution buffer.
5. The different concentrations of labeled control RNA and the test RNA were dotted 1 µl on the nitrocellulose membrane, respectively.
6. Cross-linked under ultraviolet lamp for 3 min.
7. Washed with washing buffer (100 mM Tis–HCl pH 7.5, 150 mM NaCl, 0.3%(v/v) Tween).
8. Incubated in the blocking buffer (100 mM Tis–HCl pH 7.5, 150 mM NaCl, 1% (w/v) blocking reagent) for 30 min, then in the antibody buffer 1 (diluted the Anti-DIG-AP-antibody in the blocking buffer at a ratio of 1:5000) for 30 min.
9. Washed twice in the washing buffer, 15 min each time, kept shaking gently.
10. Incubated for 2 min in the detection buffer (100 mM Tis–HCl pH 9.5, 100 mM NaCl), the Nitro-blue-tetrazolium (NBT)/5-bromo-4-chloro-3-indolyl-phosphate (BCIP) (Roche, Mannheim, Germany) solution (diluted NBT/BCIP 1:50 in 100 mM Tris pH 9.5, 100 mM NaCl, 50 mM MgCl_2_ solution) was used to determine the concentration of the test RNA.

#### Tissue fixation

The shoot apical meristems of turnip seedlings were placed in the fixative solution. Vacuumed the materials on ice for 15 min. And repeated this step several times until the materials were down to the bottom. We used two kinds of fixative methods to fix the materials in this study.

1. FAA fixative was used for fixation at 4 °C for 14 h. The mixture of 50% ethanol, 10% formaldehyde, 5% glacial acetic acid, and 35% DEPC- treated H_2_O, stored at room temperature.
2. 4% Paraformaldehyde was used for fixation at 4 °C for 14 h. The polyoxymethylene powder was added to pH 11 phosphate buffer saline (PBS) and stirred to dissolve at 60–70 °C. The mixture was added with dilute sulfuric acid to adjust the pH value to 7.

#### Tissue dehydration, transparency, waxing, and embedding

These procedures were performed as follows:

1. 50% ethanol, 60% ethanol, 70% ethanol, 85% ethanol, 95% ethanol each for 1 h, 100% ethanol 30 min (repeat again, then again twice for 1 h), all steps were performed at 4 °C.
2. 25% Histo-clear + 75% ethanol, 50% Histo-clear + 50% ethanol, 75% Histo-clear + 25% ethanol each for 30 min, 100% Histo-clear 1 h (repeat again), all steps were performed at 25 °C.
3. Samples were transferred to the new 100% Histo-clear with 1/4 volume paraffin wax and incubated overnight at 25 °C, and then added another 1/4 volume paraffin wax until melt completely and moved to 60 °C incubator for 4 h. Liquid paraffin wax overnight, changed fresh liquid paraffin twice 1 day, embedded after 3 days. Stored at 4 °C.

#### Section

The embedded materials were cut to 10 µm sections with Leica RM 2015 Microtome. Added DEPC-treated H_2_O to the adhesion slide of CITOGLAS (Citotest, Nanjing, China). And then it was placed at 42 °C. The wax strip was flattened on the slide, dried overnight at 42 °C.

#### Section pretreatment

1. The slides were put in 100% dimethylbenzene twice, each for 10 min, 100% ethanol twice each for 1–2 min, 95% ethanol, 90% ethanol, 80% ethanol, 60% ethanol, 30% ethanol, water each for 1–2 min, 2× SSC for 20 min to dewax and rehydrate.
2. Then protease K was added to the 37 °C protease buffer (100 mM Tris pH 8 and 50 mM EDTA) to make the final concentration 1 µg/ml. Two treatments were compared, incubation at 37 °C for 15 min and at 37 °C for 30 min. Stop the treatments in 2 mg/ml glycine for 2 min, washed twice with 1× PBS for 2 min, post-fix 4% paraformaldehyde for 10 min, 1× PBS twice each for 5 min.
3. Added 5.95 ml triethanolamine to 400 ml water and adjusted the pH to 8.0 with hydrochloric acid, then added 2 ml acetic anhydride and stir for 5 s, placed slides and stirred for 10 min.
4. Washed in 1× PBS twice each for 5 min, 30% ethanol, 60% ethanol, 80% ethanol, 90% ethanol, 90% ethanol each for 30 s, 100% ethanol twice, each for 30 s. The slide was placed in a humidity box containing anhydrous ethanol at 4 °C for 3–4 h.

#### Hybridization

Denatured the probe by heating to 80 °C for 3 min, and cooling on ice after centrifugation. Pipetted 100 µl of each probe in hybridization solution on each appropriate slide and recover the Parafilm. Hybrid at 52 °C for 12 h. The concentrations of the probes in the hybridization solution were 50 ng/ml, 100 ng/ml, and 200 ng/ml.

#### Washing

Two methods were used as follow:

Method one: 0.2× SSC and NTE were preheated to 55 °C and 37 °C, respectively.

1. The slides were washed at 55 °C for 60 min in 0.2× SSC.
2. Incubated twice in NTE at 37 °C for 5 min.
3. Added RNase A into NTE, the slides were incubated at 37 °C for 30 min.
4. Incubated twice in NTE at 37 °C each for 5 min.
5. Washed slides twice in 0.2× SSC at 55 °C, 60 min each time.
6. The slides were washed in 1× PBS at room temperature for 5 min. All the steps should keep shaking gently.

Method two: 0.2× SSC and NTE were preheated at 58 °C and 37 °C, respectively.

1. The slides were washed at 55 °C for 60 min in 0.2× SSC.
2. Incubated twice in NTE at 37 °C for 5 min.
3. Added RNase A into NTE, the slides were incubated at 37 °C for 30 min.
4. Incubated twice in NTE at 37 °C each for 5 min.
5. Washed slides twice in 0.2× SSC at 58 °C 60 min each time.
6. The slides were washed in 1× PBS at room temperature for 5 min. All the steps should keep shaking gently.

#### Blocking, antibody hybridization, and washing

1. The slides were shaken gently in blocking buffer (added 1% (w/v) or 2% (w/v) blocking reagent to 100 mM Tris, pH 7.5, 150 mM NaCl solution) for 60 min at room temperature.
2. Incubated in the fresh Albumin from bovine serum (BSA) solution (added 1% BSA to 100 mM Tris pH 7.5, 150 mM NaCl, 0.3% Triton X-100 solution) with gently swirling for 45 min.
3. The anti-DIG-AP-antibody was diluted 1:1000 in BSA solution. Each slide was carefully added 150 µl the diluent Anti-DIG-AP-antibody, covered with parafilm, and placed in a humidity box containing BSA solution. Incubated at room temperature for 2 h.
4. Washed in BSA solution for 4 times, 15 min each time.
5. Washed in 100 mM Tris pH 9.5, 100 mM NaCl, 50 mM MgCl_2_ solution for 3 times, 10 min each time. All the solutions should be freshly prepared, and all the steps were performed at room temperature.

#### Probe detection

NBT/BCIP was diluted 1:50 with 100 mM Tris pH 9.5, 100 mM NaCl, and 50 mM MgCl_2_ solutions. Each slide was added 200 µl the diluted NBT/BCIP and covered with parafilm. The slide was laid flat in a humidity box and kept wet by adding double distilled water. The slides were incubated at 25 °C for 3 days. Observed the probe signals were detected as dark purple staining by Leica DM1000 with DFC495.

## Results

### Fixation of plant material

**Fig. 1:**
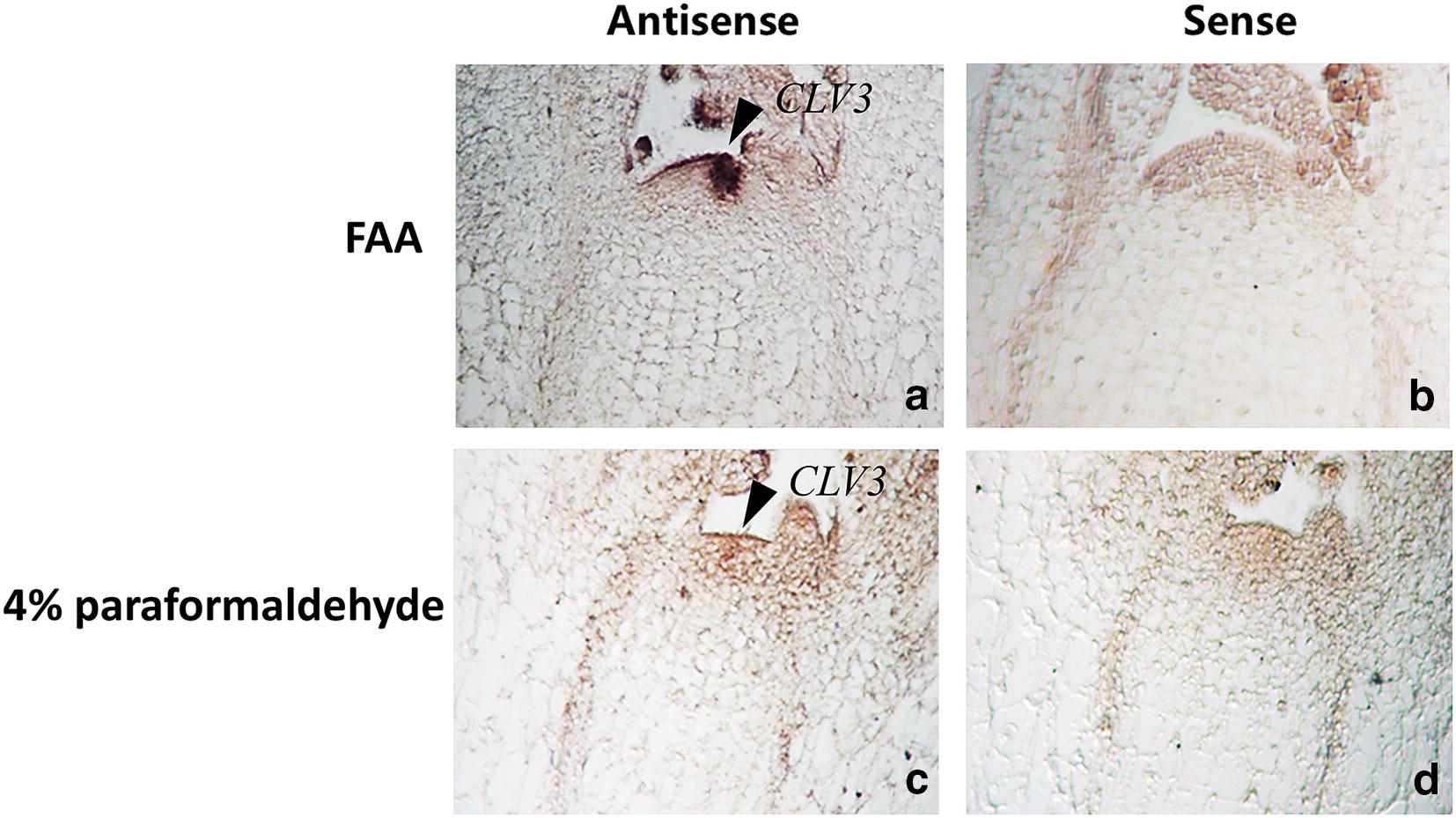
Signal and tissue morphology of *CLV3* mRNA by in situ hybridization with different fixatives. Fixing with FAA for 12 h at 4 °C lead to strong hybridized signal and integrated morphology **a** antisense, **b** sense. Fixing with 4% paraformaldehyde for 12 h at 4 °C lead to integrated morphology but weak signal intensity **c** antisense, **d** sense. Arrow, hybridized signal was found in the meristem of turnip

FAA is an optimal fixative in this protocol. 100 bp length of antisense/sense probes with 100 ng/ml were used to hybridize at 52 °C after 1 µg/ml Protease K treatment 30 min. Our results showed that the intensity of the hybridization signal of the FAA fixed material for 14 h at 4 °C was higher than that of the 4% paraformaldehyde fixed material for 14 h at 4 °C (Fig. 1).

### Protease K treatment time

**Fig. 2:**
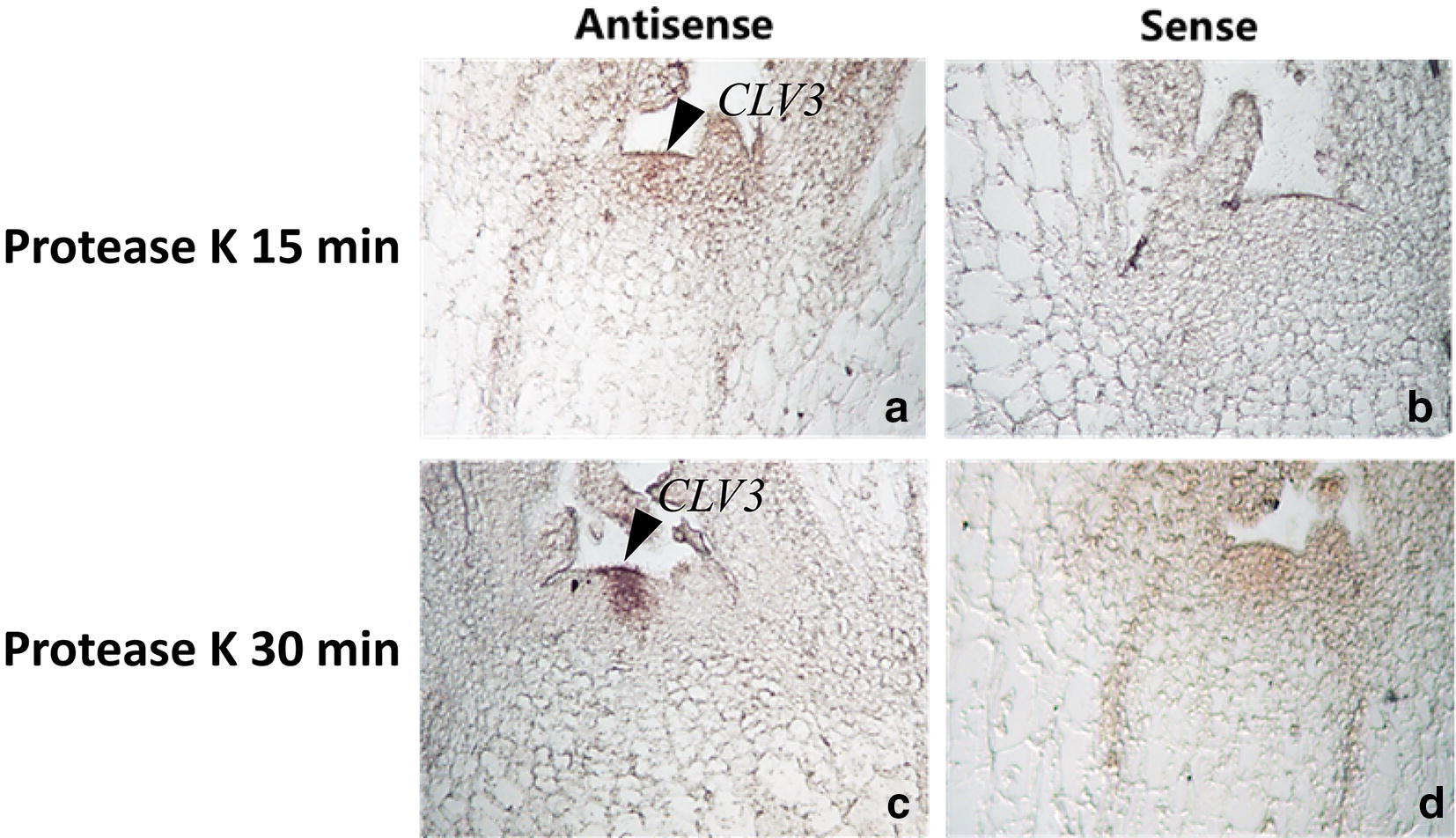
Signal of *CLV3* mRNA by in situ hybridization with different digestion time with protease K. 1 µg/ml protease K digested at 37 °C for 15 min, with weak hybridized signal **a** antisense, **b** sense. 1 µg/ml protease K digested at 37 °C for 30 min with strong hybridized signal **c** antisense, **d** sense. Arrow, hybridized signal was found in the meristem of turnip

30 min protease K treatment is recommended in this protocol. Our result shows, signals of tissues with the 30 min treatment are more intense than tissues with the 15 min treatment (Fig. 2). An appropriate protease K treatment time can be critical to receiving a satisfying signal. Theoretically, a long treatment lead facilitates probe to penetrate into the tissue well, however over-treatment will damage the tissue morphology and RNA retention that could result in the weaker signal.

### Probe length on hybridization signal

**Fig. 3:**
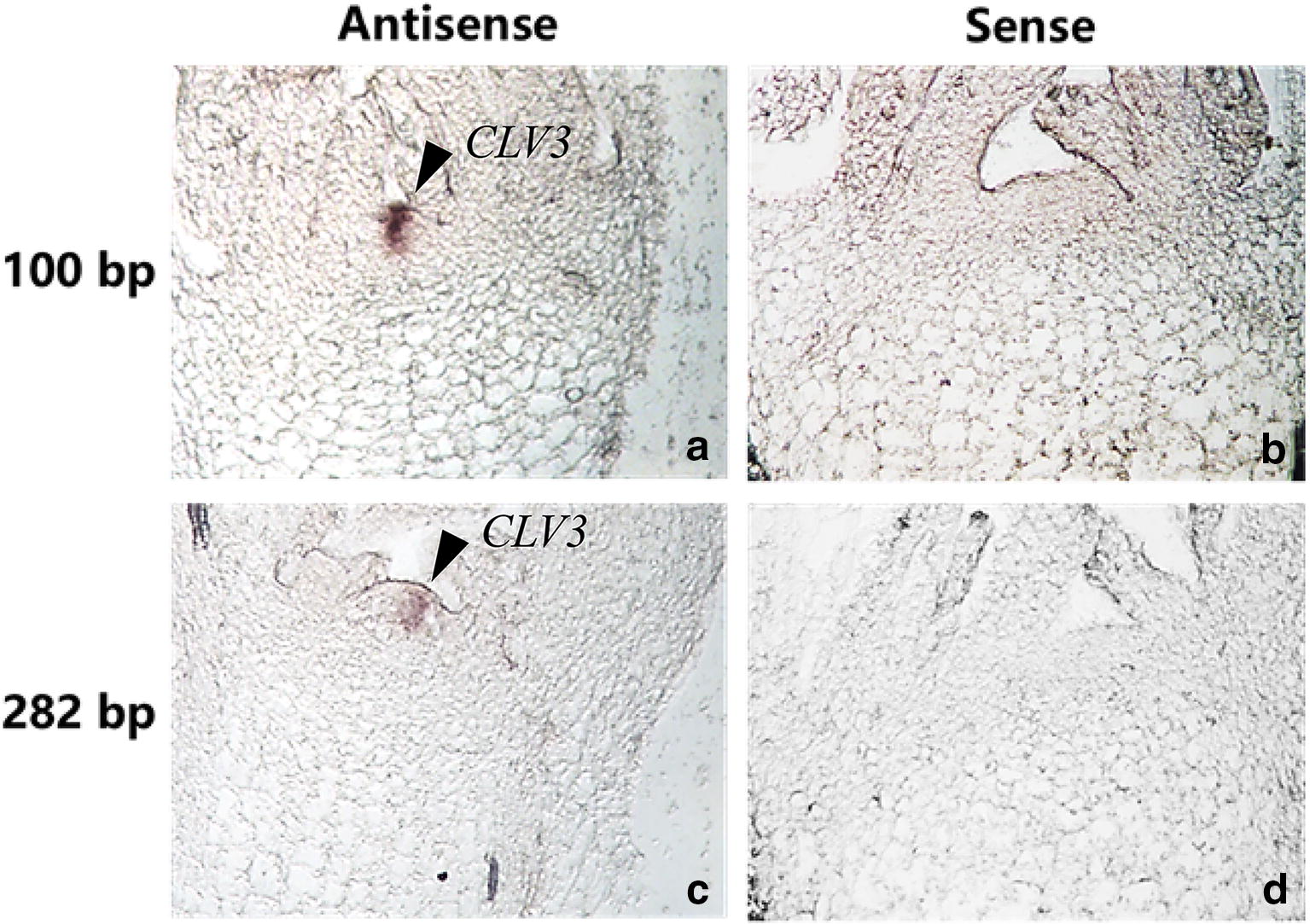
Probe length of *CLV3* mRNA on in situ hybridization signal. Hybridized with the hydrolyzed probe (100 bp) to produce strong hybridized signal. **a** Antisense, **b** sense; hybridized with the unhydrolyzed probe (282 bp) **c** antisense, **d** sense. Arrow, hybridized signal was found in the meristem of turnip

100 bp was the best probe length in this protocol. The materials were fixed by FAA for 14 h at 4 °C, digested by protease K for 30 min. Antisense/sense probes with 100 ng/ml were used to hybridize. Our results showed that signal was strong when the probe length was 100 bp and weak when the probe length was 282 bp (Fig. 3).

### Probe concentration on hybridization signal

**Fig. 4:**
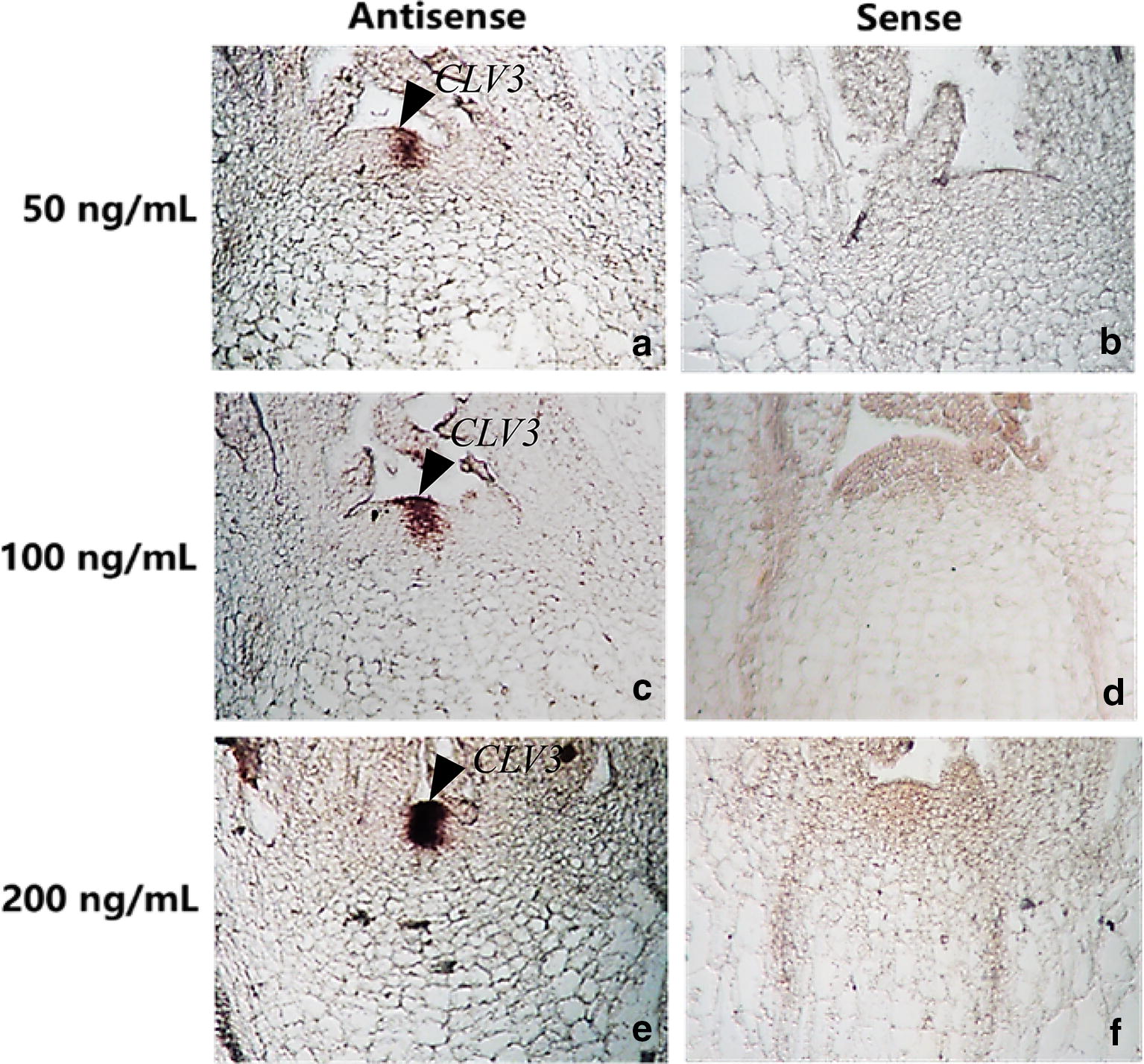
Different probe concentration of *BrrCLV3* mRNA on signal and background in situ hybridization. The concentration of the probe had no significant effect on the background dyeing. Hybridized signal: **e** 50 ng/ml > **c** 100 ng/ml > **a** 200 ng/ml. **a**, **c**, **e** antisense, **b**, **d**, **f** sense. Arrow, hybridized signal was found in the meristem of turnip

The 100 ng/ml probe was ideal concentration in this protocol. Three concentrations of probe were used to test. Our results showed that the signals were coincidence with the probe concentrations. The hybridization signal was strongest at the concentration of 200 ng/ml, followed by 100 ng/ml, and weakest at 50 ng/ml but 200 ng/ml probe give background (Fig. 4).

### The applicability of the in situ hybridization

**Fig. 5:**
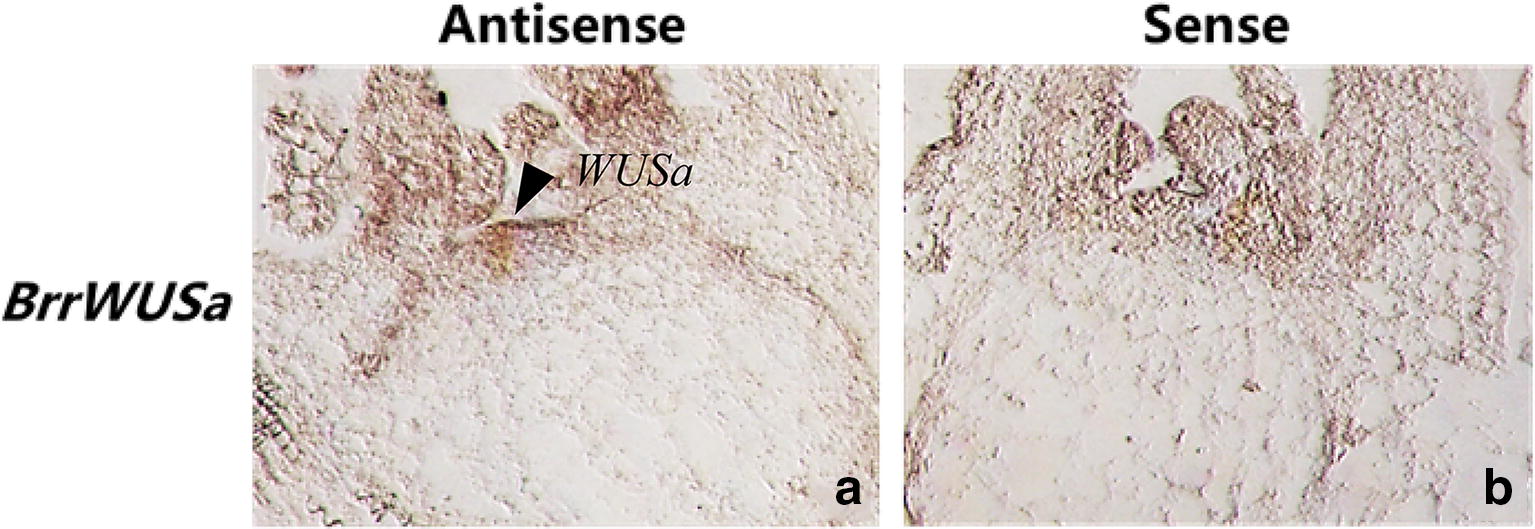
*BrrWUSa* was detected in SAM by in situ hybridization. **a** Antisense, **b** sense. Arrow, hybridized signal was found in the meristem of turnip

This protocol is optimized for turnip. The material was fixed at 4 °C for 14 h with FAA and treated with proteinase K for 30 min. The 100 ng/ml, 100 bp antisense/sense probe was hybridized at 52 °C. To test whether the established mRNA ISH system of turnip is suitable for other genes. The *BrrWUSa* was selected to determine the ISH of turnip due to the clear expression position in the meristem of turnip. Our results showed that *BrrWUSa* was specifically expressed in the apical meristem (Fig. 5).

### Background staining of mRNA ISH system

**Fig. 6:**
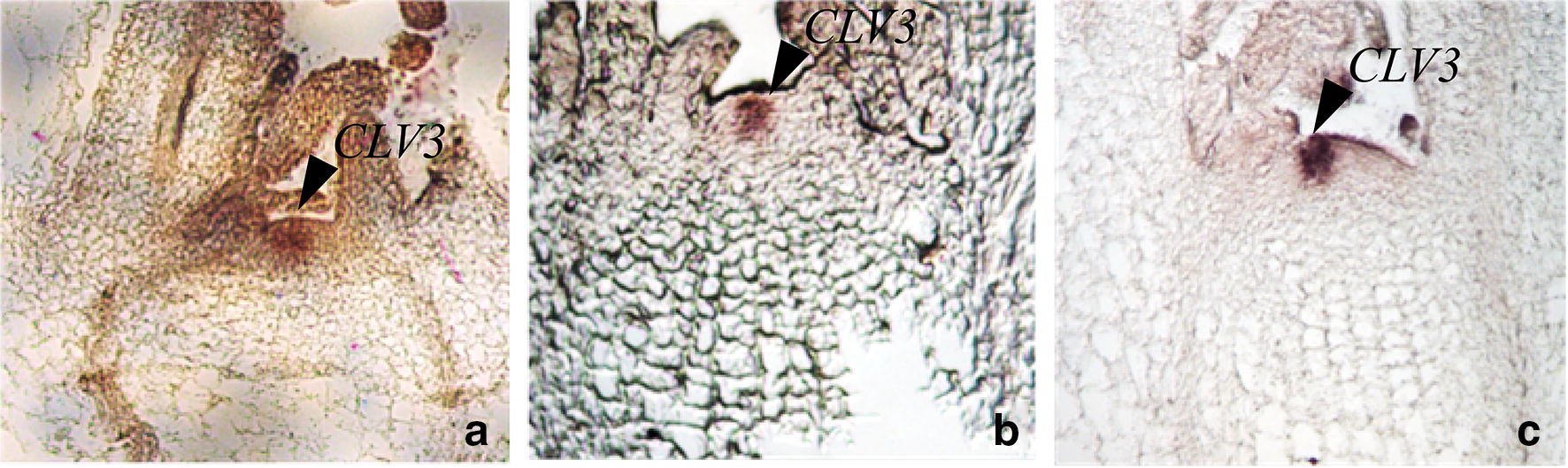
The effect of different washing methods on background and tissue morphology. **a** Washing temperature: 55 °C, washing solution concentration: 0.2× SSC; washing times: 3 times; 1% (w/v) blocking reagent; blocking time: 45 min; **b** washing temperature: 58 °C, washing solution concentration: 0.1× SSC; washing times: 5 times; 2% (w/v) blocking reagent; blocking time: 60 min; **c** washing temperature: 55 °C, washing solution concentration: 0.2× SSC; washing times: 3 times; 2% (W/V) blocking reagent; blocking time: 60 min; the washing process after hybridization was performed with sense probe and antisense probe in different staining tanks. Arrow, hybridized signal was found in the meristem of turnip

The background staining was significantly reduced, with the tissue morphology complete. As shown in Fig. 6, the washing temperature was raised from 55 to 58 °C, the wash buffer was reduced from 0.2× to 0.1× SSC, the washing times were increased from 3 to 4 times, and the concentration of blocking reagent in the blocking buffer was increased from 1 to 2% (w/v), the blocking time was increased from 45 to 60 min. It was slightly weakened the background, but the tissue morphology was damaged with the increase of washing temperature and times (Fig. 6b). The washing times increase resulted in the flaking rate increase and prolonged the experimental time. To avoid these problems, we increased the concentration of blocking reagent from 1 to 2% (w/v) and the blocking time from 45 to 60 min. The results showed that the background staining was significantly reduced, with the tissue morphology complete (Fig. 6c).

### Optimization of RNA probe transcription system

**Fig. 7:**
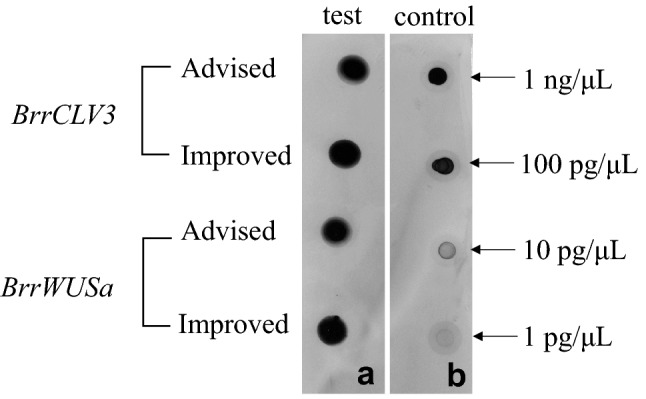
Optimization of RNA probe transcription system. RNA probe concentration was detected by dot blotting. **a** Test RNA probe, **b** control RNA probe. The transcriptional products were increased by improved method

The optimized transcription system could increase the transcriptional efficiency. To increase transcriptional efficiency, the 11.5 µl complete digestion product was used directly as the template after single-enzyme digestion. RNasin was increased from 1 to 2 µl, RNA polymerase was increased from 2 to 2.5 µl, and the incubation time was increased from 2 to 5 h. The dot blotting assay showed that the transcriptional products were significantly increased in this transcription system (Fig. 7).

## Discussion

### Best fixation method and pretreatment time in turnip’s ISH system

Tissue fixation is an important step in ISH. The most frequently used fixatives in ISH are 4% paraformaldehyde and FAA. Different fixatives have various tissue morphological integrities, mRNA retention and probe penetration [15]. Long digestion time or high concentration of protease K can damage the tissue morphology and degrade mRNA, whereas inadequate digestion can prevent the probe from penetrating the tissues [16]. The optimum concentration and treatment time of protease K were also affected by the fixation method. The concentration or treatment time of protease K should be increased when the fixation solution is strong or the fixation time is long [17, 18]. In this study, the FAA-fixed materials had stronger hybridized signals and maintained better tissue morphology than that of 4% paraformaldehyde-fixed materials. Compared with 4% paraformaldehyde, the FAA fixative was not only easier to prepare and store, but also more stability. Therefore, FAA is more suitable than 4% paraformaldehyde in the mRNA ISH of turnip. After fixation at 4 °C for 14 h with FAA, the turnip’s meristem was treated with 1 µg/ml protease K for different times. There were no hybridization signals after digestion 15 min. When digestion time was prolonged to 30 min, we observed strong hybridization signals. Therefore, the material should be incubated with 1 µg/ml protease K for 30 min in the ISH of turnip.

### Optimal probe length and concentration in turnip’s ISH system

It is theoretically believed that long probes containing more DIG labels can improve the sensitivity of hybridization, but long probe will be difficult to penetrate into the tissue. The optimum probe length is 100–300 bp. These probes can readily penetrate the tissue and stably bind to the target sequence [19]. In this study, FAA fixative was used for 14 h, and 1 µg/ml proteinase K was used for 30 min. Hybridization was performed with 282 bp prolonged probe and 100 bp probe. The hybridization signal was strong when the probe length was 100 bp. Thus, the 100 bp probe is suitable under the given fixation method and pretreatment time.

The probe concentration affects the background and the hybridization signal intensity. High probe concentrations lead to strong background [19], whereas low probe concentrations lead to weak signal [18]. The ideal probe concentration will obtain good signal. In general, the probe concentration is controlled between 100 and 600 ng/ml in ISH [20, 21]. Some researchers suggested that the optimal probe concentration is related to the probe length and should be kept at 0.5 ng/µl/kb [22]. According to this theory, the optimum concentration of 282 bp *CLV3* probe should be 141 ng/ml. In this study, the probe concentrations were set to 50, 100, and 200 ng/ml, and the results showed that the signal intensity was consistent with probe concentration. But the high probe concentration resulted in significant background, indicating that the optimal probe concentration may be between 100 and 200 ng/ml. *BrrWUSa* has a specifical expression pattern in SAM, which was used to test whether the established mRNA ISH system of turnip is adapted to the other genes of turnip. The specificity of the signal was observed in the SAM. The results indicated that this protocol is optimized for turnip.

### Means of reducing background

The background is the main problem in the ISH. Background staining can interfere researchers’ judgment, and even cover up hybridization signals. To decrease background, optimal conditions must be determined empirically, depending on washing temperature, the washing times, blocking reagent content, the blocking time and the washing solution concentration. But the increased washing temperature damaged the tissue morphology. Furthermore, the increased washing and blocking times increased the experiment time and flaking rate. The slides with sense and antisense probes were washed in different dyeing tanks after hybridization. The background decreased significantly, and remained optimal tissue morphology. It may be due to the fact that in the hybridization process, the sense probe is immersed in the slide with antisense probe by the washing buffer during washing process, which results in background. Therefore, once the antisense probe is washed separately form the sense probe, the background will be significantly weakened.

### Improvement of transcription efficiency of RNA probe in vitro

In vitro transcription of RNA system, DNA template and RNA polymerase are key factors for transcriptional efficiency. DNA extraction efficiency from gels or ethanol precipitation is too low to meet the concentration requirements of in vitro transcription system. The enzymatic digestion product was directly used as DNA transcription template to avoid recovery loss by gel extract or ethanol precipitation. Meanwhile, increasing the amount of RNasin and RNA polymerase and the incubation time will improve the transcription efficiency, thereby shortening the experimental period and reducing the experimental cost.

## Conclusions

Taken together, we established and optimized an mRNA ISH system for turnip by exploring various experimental conditions. An effective method of reducing the background staining was obtained by optimizing the factors which affect the background. Furthermore, we optimized the in vitro transcription system of the RNA probe. The difficulty and cost of the in vitro transcription experiment of the RNA probe were reduced, and the efficiency of in vitro transcription was improved. This research provides a powerful tool for studying the function and expression of genes in turnip.

## Data Availability

The datasets used and/or analyzed during the current study are available from the corresponding author on reasonable request.
